# Condition-Based Failure-Free Time Estimation of a Pump

**DOI:** 10.3390/s23041785

**Published:** 2023-02-05

**Authors:** Grzegorz Ćwikła, Iwona Paprocka

**Affiliations:** Faculty of Mechanical Engineering, Silesian University of Technology, Konarskiego 18A, 44-100 Gliwice, Poland

**Keywords:** condition-based maintenance, predictive maintenance, pump maintenance, genetic algorithm, SCADA, data acquisition, wastewater treatment plant (WWTP)

## Abstract

Reliable and continuous operation of the equipment is expected in the wastewater treatment plant, as any perturbations can lead to environmental pollution and the need to pay penalties. Optimization and minimization of operating costs of the pump station cannot, therefore, lead to a reduction in reliability but rather should be based on preventive works, the necessity of which should be foreseen. The purpose of this paper is to develop an accurate model to predict a pump’s mean time to failure, allowing for rational planning of maintenance. The pumps operate under the supervision of the automatic control system and SCADA, which is the source of historical data on pump operation parameters. This enables the research and development of various methods and algorithms for optimizing service activities. In this case, a multiple linear regression model is developed to describe the impact of historical data on pump operation for pump maintenance. In the literature, the least squares method is used to estimate unknown regression coefficients for this data. The original value of the paper is the application of the genetic algorithm to estimate coefficient values of the multiple linear regression model of failure-free time of the pump. Necessary analysis and simulations are performed on the data collected for submersible pumps in a sewage pumping station. As a result, an improvement in the adequacy of the presented model was identified.

## 1. Introduction

Company managers are currently trying to maximize profits by optimizing the use of resources and reducing costs while taking into account such criteria as ecology, environmental protection, and minimizing the carbon footprint [1]. However, there are examples of such applications in which optimization and minimization of costs, especially maintenance costs, cannot be the most important management criterion. One such example is sewage treatment plants that must operate continuously and reliably 24/7 [2]. In the event of a major failure, which involves stopping the flow and treatment of wastewater, the environment may be contaminated, and high fines may be payable [3]. Equipment in the sewage treatment plant, such as pumps, must be capable of continuous operation with high reliability [4,5]. It should also be taken into account that wastewater treatment processes are partly biological in nature, and errors in operation can lead to very-long-term consequences due to the need to obtain an appropriate bacterial flora [6,7].

The main pumping station of a wastewater treatment plant (WWTP) or water treatment plant (WTP) is usually designed as a multi-pump unit consisting of redundant systems due to the need to be flexible in terms of varying operating conditions (e.g., temperatures, depending on climate/season of the year; varying amounts of incoming wastewater, solid, and other contaminants; and chemicals, in the case of treatment of not only municipal sewage but also from industrial plants) [8,9]. In such conditions, it is necessary to minimize the maintenance costs and, at the same time, maintain the continuity of operation, already taken into consideration, in part, thanks to the redundancy of the systems [2,10]. The ability to anticipate impending failures by monitoring the available operating parameters of the pumps and performing preventive service work at a convenient time allows these criteria to be met [11,12].

Modern sewage treatment plants are now often equipped with an integrated control system built on the basis of controllers (PLC—Programmable Logic Controller and DCS—Distributed Control System) and enabling control and supervision via the SCADA (Supervisory Control and Data Acquisition) system [13]. The SCADA system is designed individually for a specific plant, allowing for integrated automatic control of all automated devices from the dispatcher’s station or locally by operator stations (the system can perform its own control algorithms, but at the same time, it allows operators to change setpoints and take over control in a specific situations), data acquisition, archiving, and reporting. The control system is designed to ensure the correct course of wastewater treatment processes, taking into account their variable composition, temperature, and inflow [14]. In such a system, detailed data from measuring devices (and about the operation of devices which are directly controlled by industrial automation systems) are usually available [13]. The SCADA system rarely allows for advanced analysis of the collected data using artificial intelligence algorithms, for example, but the data usually can be exported and processed in other applications [15,16].

The article focuses on the analysis of operating conditions and reliability of sewage pumps, which are one of the key devices that determine the uninterrupted operation of the sewage treatment plant.

### 1.1. Discussion of Existing Works

In this section, we provide a more thorough discussion of the literature items that may be useful in our project.

Methods used in condition-based and predictive maintenance can be sub-divided into three main categories: model-based methods, data-driven methods, and signal-based methods. The following paragraphs describe examples of these methods, especially in relation to pump maintenance issues.

In the article [11], the authors focused on an attempt to reduce the operating costs of devices, attempting to depart from the maintenance deadlines rigidly imposed by the pump manufacturer. It is proposed to extend the time between maintenance operations but with the option of completing them sooner, if necessary. The Risk-Based Maintenance (RBM) Strategy has been developed to balance cost, efficiency, and risk by scheduling repairs first to equipment being most at risk of failure. Data on failures of pumps, valves, pipework, switchboards, telemetry, and structures were taken into account. Failure data were obtained from the CMMS (Computerized Maintenance Management System). A fault tree and risk rating matrix were developed, which allowed a risk rating distribution to be obtained. This approach made it possible to indicate which subsystems should be subjected to preventive maintenance, depending on the MTBF and which systems can operate in Run-To-Failure (RTF) mode. Preventive maintenance and the supply of spare parts could, therefore, be planned according to the risk of failure of certain components. It can be seen that, in this case, current data from the SCADA system or sensors of the control system were not used, but the higher-level data on the occurrence of failures and repairs were used. Such systems are not able to predict failure in real-time on the basis of observation of the current state of the pump but can only statistically estimate the time of failure to prevent it or prepare for it.

Anis [12] developed a mathematical model to optimize planning decisions related to the maintenance of centrifugal pumps operating in a wastewater treatment plant (WWTP). The goal was to reduce costs resulting from either planned or unscheduled downtime for repairs. On the basis of the available data, four models were identified: hydraulic, operation, deterioration, and optimization. The main parameters taken into account are the level of liquid in the tank that the pumps were required to pump out and the planned pump operation schedule; it was also necessary to take into account the fact that the pumps could work with adjustable capacity (intermediate states of the pumps are possible, not only off or working at maximum capacity). The deterioration model made it possible to take into account the impact of wear on various components, such as seals, on pump performance.

The article [17] presents an analysis of the application of data on the operation of high-power pumps in order to identify efficiency opportunities, evaluate efficiency metrics, and assess applicability of operating space analysis. The goal was to reduce energy costs and minimize maintenance by using the most efficient pumps and matching capacity and number of pumps in operation to current demand. Datasets with hourly and 5 min sampling rates were averaged and used to calculate true weighted efficiency, demonstrating the effects of efficiency metric selection. Operating space analysis was used to evaluate pump control algorithms (i.e., control of variable speed drives), affecting the performance of a system. Data on pump status, flow, consumed power, and motor speed were collected automatically, while differential pressure data were intermittently measured by analog gauges and recorded manually. On the basis of this data, strategies to improve system efficiency using time series analysis, pump and system curve intersections, comparison of operation of different pumps, and control optimization were proposed. It has been shown that the characteristics of the pumps, identical at the beginning of operation, differ after some time due to the different degree of their wear and repair methods. Knowing these parameters allows them to be included in the control algorithms to optimize energy efficiency.

Monitoring of the motor operating parameters (especially the current) was the basis for the cloggage detection method of the pump rotor in the article [18]. The subject of the analysis were submersible pumps, often found in applications such as WWTP and WTP, to which direct access during operation is impossible. In such a situation, the ability to remotely monitor the status of devices is particularly important, which can be implemented through continuous control of selected parameters. Detection of a cloggage should allow the pump to be stopped before it becomes damaged. Due to the limitations of Motor Current Signature Analysis (MCSA), as this method does not work during start-ups or under changing load conditions, the authors decided to additionally use the Advanced Transient Current Signature Analysis (ATCSA) diagnostic method. Both of these methods are based on spectral analysis. As a result, it was possible to detect the symptoms of impending cloggage of the rotor, allowing for the appropriate response of the control system during various phases of pump operation.

The subject of analysis in the article [19] were gas turbines, but the similarity of the methodology used and the type of available data allows an attempt to apply these studies to optimize pump operation. Sensors collecting real-time data on temperatures of exhaust gas and burner tip were sources of data; an additional problem stems from the need to detect faulty sensors and to assess the number of required sensors in multi-sensor systems, allowing the machine to continue operation. Signal reconstruction techniques were also applied, which can be additionally used to detect sensor faults.

Failure analysis of the rainwater axial pumps installed in a wastewater pumping station are developed to enable service work to be carried out while minimizing unnecessary actions and, at the same time, reducing the risk that it will cause unwanted unforeseen failures [20]. In addition, device manufacturers are trying to provide better, optimized devices [21,22], ensuring long-term operation and resistance to typical hazards in this work environment [18]. Advanced control algorithms are also being developed, from the level of individual devices and their groups, to the complex control of the SCADA process at a high level—overall control of all sewage treatment processes [23]. Various modeling methods and combining theoretical models with real devices as a digital twin are also being developed [12,24,25]. Dhanraj et al. [26] provided a review of solar panel failure detection methods, the methods of fault detection, and monitoring of photovoltaic panels that can be adapted to other devices that require continuous operation and quick response to changing conditions. Methods used in reliability analysis and failure prediction include algorithms that compare parameters collected under normal operating conditions with current ones, parameter-based models, fault tree analysis, and machine learning techniques.

Summing up the review of the literature on the operation of devices, especially pumps, the following methods are distinguished along with the frequency of data collection: periodic inspections of technical condition (troublesome in the case of submerged pumps), periodic or continuous monitoring of the values normally available in control systems, as well as installing additional sensors to collect more detailed data [27].

Let us now analyze examples of maintenance methods, in particular those based on linear regression issues. In the article [19], a generalization of multi-dimensional linear regression was proposed in order to facilitate multi-sensor fault detection and signal reconstruction through the use of analytical optimization. The analytical formulation was used to propose a low computational overhead technique for real-time monitoring. Authors recommend this technique for a variety of devices that require continuous, reliable operation for safety reasons.

When developing methods for modeling and predicting pump failures in sewage treatment plants, one can also rely on the experience of other industries. Gong et al. [28] worked on a condition-based decision-making approach for the inspection time of a ship, assuming that the life-cycle–cost probability distribution contains two components. The first is the cost of a ship maintenance (distinguishing dry-docking and structural renewal), and the second is the cost of failure consequences. In this case, it is important to determine cost-effective inspection times that allow for longer operation of the ship while maintaining an acceptable level of risk and safety [28].

Girtler, in [29], proposed the three-state semi-Markov model of the process of state transitions of a ship’s main engine with three states: full serviceability, partial serviceability, and unserviceability. Empirical data concerning the engine were used for calculating limiting probabilities for the process, which can be used in decision making with the support of Bayesian statistical theory.

Wu et al. [30] developed a component maintenance priority measure used to select additional components for preventive maintenance (PM) when performing PM on critical component of a ship. In article [31], the author proposed the use of regression analysis to control the level of maintenance of the ship’s main engines. Factors taken into account when determining the level of maintenance were, among others, the weight of the element, the number of structural connections, and the operating temperature of the element. Regression functions were also used for the piston and crankshaft system, fuel system, cooling and lubrication systems, turbocharging system, and the starting system, with high multiple determination coefficients.

The authors of article [32] focused on the subject of shortening the time of the ship’s stay in the ship repair yard. This stay should be minimized, and the authors used a multiple linear regression model in which the ship’s repair time was a function of the ship’s age, deadweight, and previous repairs to the paint coating of the hull, tanks, and structural steel. Method of least squares was applied to estimate the regression coefficients. Multiple linear regression analysis were also adopted from this article by the authors of [33] in order to assess the ship’s maintenance needs. The authors proposed a genetic algorithm (GA) to determine the coefficient values of the multiple linear regression model, and the analyses were carried out on data from ships intended for the transport of oil and chemicals. Improved algorithms from this publication have been adapted to predict the time of correct operation of pumps in a sewage treatment plant.

The literature review shows that even though there are some articles on pumps control and maintenance optimization [12,17,20], only a few address the problem of estimating the failure-free time of a pump of the sewage treatment plant by observing the relationship among the data on pump condition and environment and failure-free time. This paper presents an approach to estimating the failure-free time for managers to assist in advance maintenance planning. The presented approach was also applied to find a hidden pattern that shows the relationship between maintenance work and maintenance duration. The original value of this paper is the application of a multiple linear regression model to a new problem: failure-free time estimation of a pump in a sewage treatment plant.

### 1.2. Goals and Approaches

One of the managers’ goals is to pre-define the scope of maintenance works on a critical infrastructure. Another goal is to estimate the duration of maintenance and failure-free times in order to build predictive maintenance schedules and plan the workload of maintenance workers [34]. The purpose of this paper is to develop a model to predict a pump maintenance time. A multiple linear regression model is used to describe the effect of tank level (m), power (kW), voltage (V), frequency (Hz), current (A), and torque (Nm) on the failure-free time of a pump. The linear function with the highest value of the multiple determination coefficient and the smallest standard deviation value is sought. Data on operation of pumps are collected from SCADA system at sewage treatment plants in a region with a relatively high population density and concentration of industrial plants.

Another objective is to identify variables that have a significant impact on the pump’s failure-free time and their inter-relationships. The objective of the paper is to present a condition-based prediction model to evaluate the failure-free time considering historical data.

The use of a GA to estimate values of linear regression coefficients describing the relationship between the recorded data on the pump condition and the time of failure-free operation is the original part of this article.

The paper is organized as follows: Section 2 contains characteristics of the pump station in the investigated sewage treatment plant; the next Section 3 presents a model for estimating the duration of a repair, as well as the GA for estimation of the regression coefficients. Regression coefficients estimation is presented in Section 4, including application of the Solver appendix of MS Excel (Section 4.1) and proposed GA (Section 4.2). Section 5 contains further analyses and experimental test results related to the research on the application of the GA. The paper concludes with a brief summary of the results (Section 6).

## 2. Characteristics of the Pump Station in the Sewage Treatment Plant

The wastewater treatment plant (WWTP) in which the analyzed pumps operate is modern and relatively new (built in 2002, modernized in 2013). It is equipped with an integrated automatic control system based on 8 PLCs, a SCADA (Supervisory Control and Data Acquisition) system, and a fieldbus fiber optic network. This is to ensure minimization of employment with simultaneous optimal and safe operation, allowing for obtaining a legal level of wastewater treatment.

A WWTP is an example of an installation that must operate continuously, 24 h a day, 7 days a week, all year round. The WWTP staff works in three shifts and consists of the management staff (manager and chief technologist, who work only on the first shift), dispatchers (one on each shift, who are responsible for continuous supervision of the sewage treatment plant operation via the SCADA system and overview of operators and maintenance workers), teams of operators working in individual shifts (responsible for ongoing operation of processes, equipment supervision, auxiliary and transport work, and removing minor problems), and maintenance workers (mechanics, electricians, and automation specialists, responsible for solving emerging problems as well as carrying planned maintenance works).

Working in a WWTP is associated with many inconveniences and dangers, including unpleasant odors, the risk of falling into open tanks and installations, microbiological hazards, dangerous gases, and the risk of explosion (in some wastewater treatment processes, methane and other gases are byproducts that can be even used to generate heat and electricity) [6,10].

Due to the need for continuous operation and changing conditions, including the amount and composition of wastewater (variable proportions of municipal and industrial sewage), sewage temperature, and ambient temperature, a large part of the key equipment and installations is redundant, and there are spare devices and connections between/among the sewage treatment plant departments/modules. Data adopted at the design stage of the WWTP include: average daily sewage flow (Q_dśr_) 51,000 m^3^/day, maximum daily flow (Q_dmax_) 60,000 m^3^/day, maximum hourly flow during rainless weather (Q_hmax_) 3825 m^3^/h, and maximum hourly flow during rainy weather (Q_hmax-max_) 5988 m^3^/h.

Wastewater is treated in the first stage by mechanical processes (draining and sedimentation) and in the second stage biologically (wastewater is processed by microorganisms that use it as food and bind various chemical compounds, which then must be removed) [2]. Biological processes require very precise regulation of conditions (temperature and time/flow rate), and any deviations may lead to the death of the favorable microorganisms and possibly the development of unfavorable ones. The variability of wastewater composition and temperatures means that the reactors must be precisely dosed with appropriate auxiliary substances, which is the responsibility of wastewater composition sensors, control system, technologist, and dispatcher. Obtaining and maintaining the right bacterial flora is a very difficult and slow process, and mistakes can have long-term effects (the operation of the WWTP can be disturbed for months or even years, leading to additional costs, environmental pollution, and the need to pay penalties).

The high variability of many factors influencing the way the WWTP works means that even an advanced automatic control system must be supervised and supported by a technologist and dispatcher, and many technological installations and devices require partly manual operation by operators (e.g., cleaning grates from large objects that flowed with sewage).

The raw wastewater pumping station (RWPS) is part of the WWTP, located at the very beginning of the technological scheme. It is directly supplied by wastewater from the sewage system of a medium-sized city (population of approximately 170,000 people and many industrial plants producing wastewater with a different composition than typical household wastewater) in a Central European country with a temperate climate.

The main task of the RWPS is to lift sewage (inflowing by gravity through underground channels) to a height of approximately 6 m above ground (level 0), due to which in the next stages of sewage treatment, wastewater flow is gravitational—it does not have to be additionally forced. A complete shutdown of the pump station is practically impossible, as it would stop the wastewater treatment processes, which could lead not only to disturbances in biological treatment processes but also to an increase in the level of wastewater in sewer collectors (and in extreme cases, to the need to discharge wastewater and its pollution to the river). It can be seen that the availability of efficient, ready-to-work pumps is crucial for maintaining the continuous operation of WWTP so as not to lead to an ecological disaster and the need to pay high penalties.

### Description of the Main Raw Sewage Pumping Station

Wastewater is supplied to the RWPS by three main sewage collectors: the main sewage collector with a diameter of 1.5 m, supplying wastewater from the city center and the industrial district; an additional collector with a diameter of 0.8 m, supplying wastewater from new districts; and a combined collector with a diameter of 1.5 m, supplying combined wastewater and rainwater from city districts without separate collectors. The supply collectors connect in the inlet chamber, where the flow is equalized and the kinetic energy of the wastewater is reduced. From the inlet chamber, wastewater flows to Chambers A and B of the RWPS through pipes with a diameter of 0.8 m through large mesh gratings (the gratings are designed to retain large impurities that may have entered the sewage system). These connections can be closed as needed. The main part of RWPS is a recessed below-ground-level, reinforced concrete covered tank with dimensions A × B × H 5 × 12 × 10 m.

**Figure 1:**
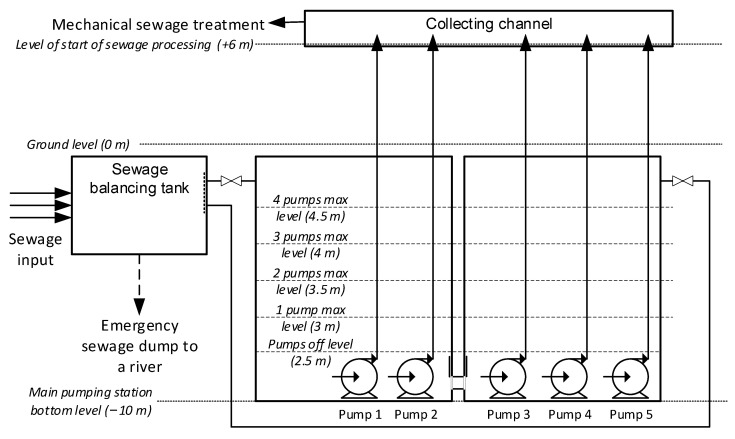
Technological diagram of the main pump station of the WWTP.

The pump station is equipped with five main pumps, installed in two chambers, A and B (Figure 1). The design parameters of the pumps are flow Q = 1500 m^3^/h and water lifting height 16.6 m. Each pump pumps wastewater through an independent pipeline to the channel in the sieve station (which is the first stage of mechanical wastewater treatment). The collector supplying wastewater from the sewage system is separated to feed both tanks with submersible pumps, and the flow of wastewater into Chambers A and B is independently controlled by valves. At the sewage inlets to the tanks, there are also gratings, which stop large-sized solid impurities/objects that could damage valves or pumps.

Chamber A has two pumps (Pumps #1 and #2) and Chamber B has three pumps (#3, #4, and #5). The subject of interest are the pump’s motors (1 to 4) controlled by inverters, allowing for their speed to be set and, consequently, for efficiency. Pump #5 is an auxiliary, controlled by a softstarter (unable to control rotation speed of the pump), and is used in exceptional circumstances (e.g., control system failure and no other pumps available) and is, therefore, not included in the analysis. All pumps are submerged, and the level of wastewater in the tank must exceed the height of the pumps by a safe margin in order for the control system to be able to start them.

The two chambers, A and B, equipped with independent wastewater level sensors are connected to each other under normal operating conditions, and the level of wastewater in them should be identical. If it is necessary to perform service work on one of the chambers, it is necessary to cut off the chamber in which the given pump is installed (closing the inflow from the collector and the connections between the tanks) and empty/dry the chamber completely. At this time, the WWTP operates on the basis of the remaining active tank and the pumps installed in it. In the event that Tank B needs to be emptied, the backup Pump #5 becomes unavailable, which reduces the safety level of the entire RWPS. The pump repair procedure is very troublesome and expensive, so there is a need to develop methods and procedures to minimize its occurrence.

Pumps in RWPS are submersible pumps (wet well installation) designed for efficient pumping of clean water, surface water, and wastewater containing solids or long-fibered material [35]. The maximum depth of immersion is 20 m. Each pump consists of two main modules: centrifugal pump (Xylem Flygt N 3356 submersible self-cleaning pump for wastewater and water 350/350 mm, power rating 45–140 kW) and motor (Xylem Flygt 665.000 motor, 3–50 Hz, 90 kW, 985 rpm, current 180 A).

Pumps are equipped with sensors: thermal sensors in the stator windings (to prevent overheating), an analog temperature sensor (to monitor the lower bearing), and leakage sensors in the stator housing/leakage chamber and the junction box. The sensors decrease the risk of bearing and stator failure.

Under average operating conditions of the WWTP, the capacity of one or a maximum of two pumps is sufficient, but it is not always necessary to use their full capacity. Simultaneous activation of all pumps may be necessary only in the event of the maximum design sewage inflow, which has been determined by taking into account the increase in the number of people and industrial plants discharging sewage.

Each of the main pumps (#1–#4) is connected to a separate inverter, enabling electronic regulation of its rotation and, consequently, its efficiency. Inverters allow the pumps to be supplied with AC of the appropriate frequency, current, and voltage and the continuous monitoring of these parameters. Each pump and inverter are also equipped with various sensors to detect unusual conditions and events, e.g., overloading or overheating. Activation of the protections stops the pump operation and sends an appropriate alarm to the control system. Stopping the pump is also possible after pressing the “emergency stop” button by the pump station operator or any person in its vicinity. The pumps can be started by the inverter in manual or automatic mode, depending on the control system of the entire WWTP (SCADA). In the structure of the control system, the inverters are connected to the local PLC.

For further analysis, in order to develop a methodology for forecasting pump failures, reports from the SCADA system were used because only such data can be obtained automatically and was allowed for us to use. More detailed maintenance data can be found in dispatchers’ work reports, but they are not available in electronic form, are often incomplete, and the format is not fully standardized.

The next section presents a model for estimating the failure-free operation of the pump as well as the genetic algorithm for determining of the regression coefficients.

## 3. Failure-Free Time Estimation Model

The problem of estimating the failure-free time of the main pump taking into account the condition data of tank level (m), power (kW), voltage (V), frequency (Hz), current (A), and torque (Nm) is investigated. In order to establish the relationship between modes and failure-free times (min), historical data are collected both for dependent (failure-free time) and independent variables (health conditions of the pump). Multiple linear regression analysis is adopted from [32] to identify the mathematical relationship between modes and failure-free times. A multiple linear regression analysis is performed for maintenance time estimation in [36].

The multiple linear regression model is presented below (1):

(1) $$b_{0}+b_{1}\cdot m_{i}+b_{2}\cdot {\mathrm{Pr}}_{i}+b_{3}\cdot {\mathrm{Ve}}_{i}+b_{4}\cdot {\mathrm{Fy}}_{i}+b_{5}\cdot {\mathrm{Ct}}_{i}+b_{6}\cdot {\mathrm{Te}}_{i}={\mathrm{FT}}_{i}$$

where

b_0_, b_1_, …, b_k_—regression coefficients; $j\in \left[1,2,\ldots ,k\right]$; k—size of independent variables (k = 6, namely m_i_, Pr_i_, Ve_i_, Fy_i_, Ct_i_, and Te_i_); FT_i_—dependent variable; $i\in \left[1,2,\ldots ,n\right]$; n—sample size (e.g., n = 54 for the main pump observed during 10 days, n = 75 for the first and third pump observed during 5 days); m_i_—level of liquid; Pr_i_—power; Ve_i_—voltage; Fy_i_—frequency; Ct_i_—current; Te_i_—torque; and FT_i_—failure-free time.

Using the least squares method [32,33,37], the objective function is achieved:

(2) $$n\cdot b_{0}+b_{1}\overset{n}{\underset{i=1}{\sum }}m_{i}+b_{2}\overset{n}{\underset{i=1}{\sum }}{\mathrm{Pr}}_{i}+b_{3}\overset{n}{\underset{i=1}{\sum }}{\mathrm{Ve}}_{i}+b_{4}\overset{n}{\underset{i=1}{\sum }}{\mathrm{Fy}}_{i}+b_{5}\overset{n}{\underset{i=1}{\sum }}{\mathrm{Ct}}_{i}+b_{6}\overset{n}{\underset{i=1}{\sum }}{\mathrm{Te}}_{i}=\overset{n}{\underset{i=1}{\sum }}{\mathrm{FT}}_{i}$$

under the constraints:

(3) $$\begin{array}{c} b_{0}\overset{n}{\underset{i=1}{\sum }}m_{i}+b_{1}\overset{n}{\underset{i=1}{\sum }}m_{i}^{2}+b_{2}\overset{n}{\underset{i=1}{\sum }}m_{i}\cdot {\mathrm{Pr}}_{i}+b_{3}\overset{n}{\underset{i=1}{\sum }}m_{i}\cdot {\mathrm{Ve}}_{i}+b_{4}\overset{n}{\underset{i=1}{\sum }}m_{i}\cdot {\mathrm{Fy}}_{i}+b_{5}\overset{n}{\underset{i=1}{\sum }}m_{i}\cdot {\mathrm{Ct}}_{i}+b_{6}\overset{n}{\underset{i=1}{\sum }}m_{i}\cdot {\mathrm{Te}}_{i}=\\ \overset{n}{\underset{i=1}{\sum }}m_{i}\cdot {\mathrm{FT}}_{i} \end{array}$$

(4) $$\begin{array}{c} b_{0}\overset{n}{\underset{i=1}{\sum }}{\mathrm{Pr}}_{i}+b_{1}\overset{n}{\underset{i=1}{\sum }}{\mathrm{Pr}}_{i}\cdot m_{i}+b_{2}\overset{n}{\underset{i=1}{\sum }}{\mathrm{Pr}}_{i}^{2}+b_{3}\overset{n}{\underset{i=1}{\sum }}{\mathrm{Pr}}_{i}\cdot {\mathrm{Ve}}_{i}+b_{4}\overset{n}{\underset{i=1}{\sum }}{\mathrm{Pr}}_{i}\cdot {\mathrm{Fy}}_{i}+b_{5}\overset{n}{\underset{i=1}{\sum }}{\mathrm{Pr}}_{i}\cdot {\mathrm{Ct}}_{i}+b_{6}\overset{n}{\underset{i=1}{\sum }}{\mathrm{Pr}}_{i}\cdot {\mathrm{Te}}_{i}=\\ \overset{n}{\underset{i=1}{\sum }}{\mathrm{Pr}}_{i}\cdot {\mathrm{FT}}_{i} \end{array}$$

(5) $$\begin{array}{c} b_{0}\overset{n}{\underset{i=1}{\sum }}{\mathrm{Ve}}_{i}+b_{1}\overset{n}{\underset{i=1}{\sum }}{\mathrm{Ve}}_{i}\cdot m_{i}+b_{2}\overset{n}{\underset{i=1}{\sum }}{\mathrm{Ve}}_{i}\cdot {\mathrm{Pr}}_{i}+b_{3}\overset{n}{\underset{i=1}{\sum }}{\mathrm{Ve}}_{i}^{2}+b_{4}\overset{n}{\underset{i=1}{\sum }}{\mathrm{Ve}}_{i}\cdot {\mathrm{Fy}}_{i}+b_{5}\overset{n}{\underset{i=1}{\sum }}{\mathrm{Ve}}_{i}\cdot {\mathrm{Ct}}_{i}+b_{6}\overset{n}{\underset{i=1}{\sum }}{\mathrm{Ve}}_{i}\cdot {\mathrm{Te}}_{i}\\ =\overset{n}{\underset{i=1}{\sum }}{\mathrm{Ve}}_{i}\cdot {\mathrm{FT}}_{i} \end{array}$$

(6) $$\begin{array}{c} b_{0}\overset{n}{\underset{i=1}{\sum }}{\mathrm{Fy}}_{i}+b_{1}\overset{n}{\underset{i=1}{\sum }}{\mathrm{Fy}}_{i}\cdot m_{i}+b_{2}\overset{n}{\underset{i=1}{\sum }}{\mathrm{Fy}}_{i}\cdot {\mathrm{Pr}}_{i}+b_{3}\overset{n}{\underset{i=1}{\sum }}{\mathrm{Fy}}_{i}\cdot {\mathrm{Ve}}_{i}+b_{4}\overset{n}{\underset{i=1}{\sum }}{\mathrm{Fy}}_{i}^{2}+b_{5}\overset{n}{\underset{i=1}{\sum }}{\mathrm{Fy}}_{i}\cdot {\mathrm{Ct}}_{i}+b_{6}\overset{n}{\underset{i=1}{\sum }}{\mathrm{Fy}}_{i}\cdot {\mathrm{Te}}_{i}\\ =\overset{n}{\underset{i=1}{\sum }}{\mathrm{Fy}}_{i}\cdot {\mathrm{FT}}_{i} \end{array}$$

(7) $$\begin{array}{c} b_{0}\overset{n}{\underset{i=1}{\sum }}{\mathrm{Ct}}_{i}+b_{1}\overset{n}{\underset{i=1}{\sum }}{\mathrm{Ct}}_{i}\cdot m_{i}+b_{2}\overset{n}{\underset{i=1}{\sum }}{\mathrm{Ct}}_{i}\cdot {\mathrm{Pr}}_{i}+b_{3}\overset{n}{\underset{i=1}{\sum }}{\mathrm{Ct}}_{i}\cdot {\mathrm{Ve}}_{i}+b_{4}\overset{n}{\underset{i=1}{\sum }}{\mathrm{Ct}}_{i}\cdot {\mathrm{Fy}}_{i}+b_{5}\overset{n}{\underset{i=1}{\sum }}{\mathrm{Ct}}_{i}^{2}+b_{6}\overset{n}{\underset{i=1}{\sum }}{\mathrm{Ct}}_{i}\cdot {\mathrm{Te}}_{i}\\ =\overset{n}{\underset{i=1}{\sum }}{\mathrm{Ct}}_{i}\cdot {\mathrm{FT}}_{i} \end{array}$$

(8) $$\begin{array}{c} b_{0}\overset{n}{\underset{i=1}{\sum }}{\mathrm{Te}}_{i}+b_{1}\overset{n}{\underset{i=1}{\sum }}{\mathrm{Te}}_{i}\cdot m_{i}+b_{2}\overset{n}{\underset{i=1}{\sum }}{\mathrm{Te}}_{i}\cdot {\mathrm{Pr}}_{i}+b_{3}\overset{n}{\underset{i=1}{\sum }}{\mathrm{Te}}_{i}\cdot {\mathrm{Ve}}_{i}+b_{4}\overset{n}{\underset{i=1}{\sum }}{\mathrm{Te}}_{i}\cdot {\mathrm{Fy}}_{i}+b_{5}\overset{n}{\underset{i=1}{\sum }}{\mathrm{Te}}_{i}\cdot {\mathrm{Ct}}_{i}+b_{6}\overset{n}{\underset{i=1}{\sum }}{\mathrm{Te}}_{i}^{2}\\ =\overset{n}{\underset{i=1}{\sum }}{\mathrm{Te}}_{i}\cdot {\mathrm{FT}}_{i} \end{array}$$

The collected data on m_i_, Pr_i_, Ve_i_, Fy_i_, Ct_i_, Te_i_, and FT_i_ is substituted in the Equations (2)–(8) simultaneously. Regression coefficients are estimated using the improved GA and Excel. The objective of the model is the selection of variables that have the greatest impact on the failure-free time. The adequacy of the presented model is computed using:

(1) a standard deviation

(9) $$s={\left[\frac{1}{n-\left(m+1\right)}\sum_{i=1}^{n}{\left(x_{i}\;-\;{\hat{x}}_{i}\right)}^{2}\right]}^{0.5},\;i\in \left[1,\ldots ,n\right]$$

(2) a multiple determination coefficient

(10) $$R^{2}=\frac{\sum_{i=1}^{n}{\left({\hat{x}}_{i}-{\overset{¯}{x}}_{i}\right)}^{2}}{\sum_{t=1}^{n}{\left(x_{i}-{\overset{¯}{x}}_{i}\right)}^{2}},R^{2}\in \left[0,1\right]$$

where: x_i_—actual value of failure-free time X in period i, ${\hat{x}}_{i}$—theoretical value of the X variable resulting from the Equation (1) in period i, ${\bar{x}}_{i}$—mean value of the variable X in the time series of length n, k—number of variables explaining the model, and k + 1—number of model parameters.

The multiple determination coefficient measures the compliance of the trend function with historical data on m_i_, Pr_i_, Ve_i_, Fy_i_, Ct_i_, Te_i_, and FT_i_. To estimate the mean time to failure, the function with the highest value of the multiple determination coefficient (10) and the lowest value of standard deviation (9) is selected.

In order to estimate mean time to failure, the GA was elaborated, including five modules: data interface, chromosomes coding, optimization, selection of chromosomes, and chromosomes decoding.

In GA, a chromosome is represented by a vector of genes, which codes the regression coefficient values for the problem of estimation of a failure-free time of a pump, while a fitness function is used to evaluate the chromosome. The fitness function measures the adequacy of the presented model to historical data describing the condition of the pump. The fitness function of a chromosome (11) is the scalar function of the coefficient of multiple determination (10) and standard deviation (9):

(11) $$F\left(x_{\eta }\right)=0.5\cdot \left(1-\frac{R{\left(x_{\eta }\right)}^{2}}{R{\left(x^{*}{}_{\eta }\right)}^{2}}\right)+0.5\cdot \frac{s\left(x_{\eta }\right)}{s\left(x_{\eta }^{*}\right)}$$

where $s\left(x_{\eta }^{*}\right)$—the maximal standard deviation achieved in a set of chromosomes in an iteration and $R\left(x^{*}{}_{\eta }\right)$—the maximal coefficient of multiple determination achieved for chromosome $x_{\eta }$.

**Figure 2:**
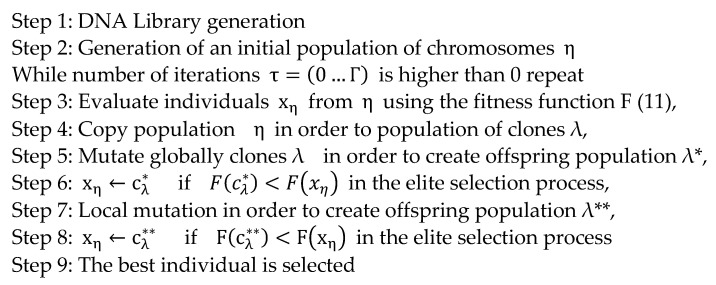
The pseudo code of the genetic algorithm.

The pseudo code of the GA is presented in Figure 2. The steps of the GA are explained below.

**Figure 3:**
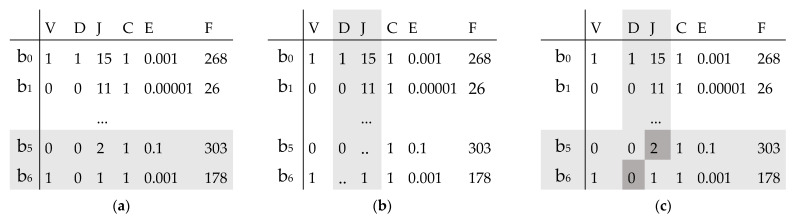
An example of a two-point mutation procedure, (**a**) parent (selected sub-chromosomes in grey), (**b**) clone (selected genes in grey), (**c**) mutated clone (nucleotide in grey).

In the condition-based estimation problem of the failure-free time of the pump, a chromosome is represented by a binary and integer-based representation. The complexity of the problem presented requires sophisticated coding practice for a chromosome, including not only regression coefficient values but also a positive or negative sign before each coefficient. Each sub-chromosome is created by random generation of the six genes from the DNA library (Table 1). Each chromosome of the individual represents a binary gene for a sign, an integer gene for a number of zeros after the decimal point for a coefficient value, and a mixed binary and integer value for a coefficient value. Each chromosome is represented by a set of sub-chromosomes, one sub-chromosome for one regression coefficient (Figure 3). By decoding each sub-chromosome from left to right, values of regression coefficients b_0_,..b_6_ are substituted into Equation (1).

The process of coding a chromosome into a multiple linear regression equation consists of coding each sub-chromosome, i.e., each regression coefficient. Consider the first row of the matrix x_j,n_ (Table 2), which encodes the regression coefficient b_0_. The genes of each sub-chromosome are presented in columns of the matrix x_j,n_. The genes are coded from left to right. The first gene of sub-chromosome b_0_ encodes the “+” sign, the second gene encodes the multiplier for the value before the decimal point (0), and the third gene encodes the coefficient value before the decimal point, that is 12. Gene (C) encodes the “0 or 1” value (Table 1), A necessary condition is added in order to generate a feasible solution: if D = 0, Gene C can only obtain the value 1, otherwise, when D = 1, Gene C can receive 1 for Sub-chromosome b_0_ (Table 2). Gene E encodes the number of zeros after the decimal point, that is, 0.0001. Gene F encodes the coefficient value after zeros, that is, 161.

For each sub-chromosome, the following decoding procedure is applied:

(12) $$x_{j,n}=\left(V\right)\cdot \left(D\cdot J+\left(C\cdot \left(E\cdot F\right)\right)\right)$$

Value 12.161 (13) is achieved for the first sub-chromosome and substituted into Equations (2)–(8) for the regression coefficient b_0_; value -12 is achieved for the second sub-chromosome, that is, for the regression coefficient b_1_, etc.

(13) $$x_{0,n}=\left(V\right)\cdot \left(D\cdot J+\left(C\cdot \left(E\cdot F\right)\right)\right)=-1\cdot \left(1\cdot 12+1\cdot \left(0.001\cdot 161\right)\right)=12.161$$

An initial population is generated by a random generation of regression coefficients (Step 2 of the GA). The parent pool is generated by copying the initial set of regression coefficients (Step 4 of the GA). A new chromosome is generated by the two-point mutation procedure in the solution differentiation process. Two sub-chromosomes and genes are randomly selected for the mutation procedure.

The mutation procedure (Step 5 of the GA) begins with the selection of two sub-chromosomes from the parent chromosome (Figure 3a). Next, one gene is selected in each sub-chromosome from V, D, J, C, E, or F. The selected genes are removed from the sub-chromosomes (Figure 3b). Offspring chromosome is produced by copying the remaining genes from the corresponding positions of the parent chromosome. The missing genes are randomly selected from the DNA library (Figure 3c). For example, Sub-chromosomes b_5_ and b_6_ are selected for the mutation procedure (Figure 3a), Genes J and D are selected for b_5_ and b_6_, respectively (Figure 3b). The selected genes are removed from the chromosome of the parent, and remaining genes are copied in the corresponding positions of the offspring’s chromosome (Figure 3b). The missing offspring sub-chromosome genes are replaced with genes randomly selected from the DNA library (Figure 3c).

For each chromosome, the decoding procedure is applied by computing the multiple regression equation (2) under the constraints (3–8). Then, in the decoding procedure in Steps 3, 6, and 8 of the GA (Figure 2), the fitness function (11) with the components of multiple determination (10) and standard deviation (9) is computed. In the elite selection procedures (Steps 6 and 8 of the GA), the best chromosome survives to the next generation of two: parent and child.

**Figure 4:**
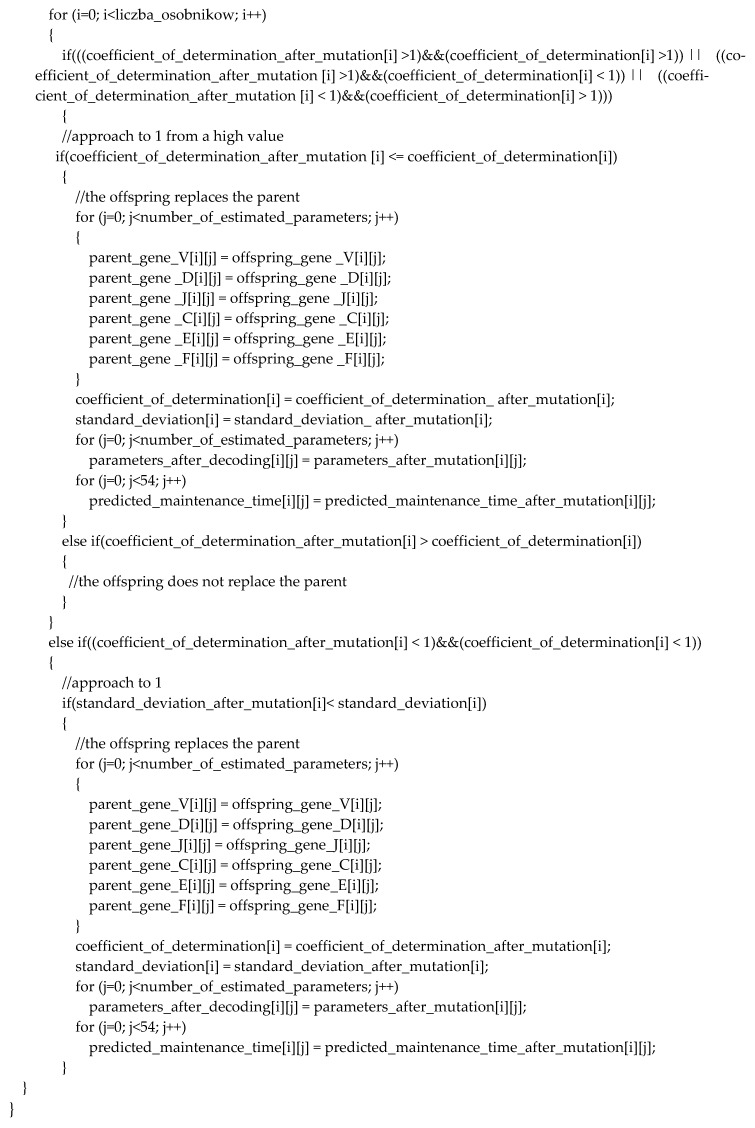
The elite selection procedure.

In Step 7, a point mutation is performed on the parent chromosome. Only the F gene on a randomly selected sub-chromosome is mutated in the point mutation procedure. The selected gene is removed from the offspring’s sub-chromosome. The removed gene is replaced with a randomly selected gene from the F library. The elite selection procedure is presented in detail in Figure 4. The best chromosome survives to the next generation of the two: parent and offspring.

A termination condition is running a predefined number of iterations. The best chromosome, which is said to be the optimal or close to the optimal, is stored in the last iteration.

The next section presents the estimation of the regression coefficients, including the application of MS Excel Solver and the proposed GA.

## 4. Regression Coefficient Estimation

**Figure 5:**
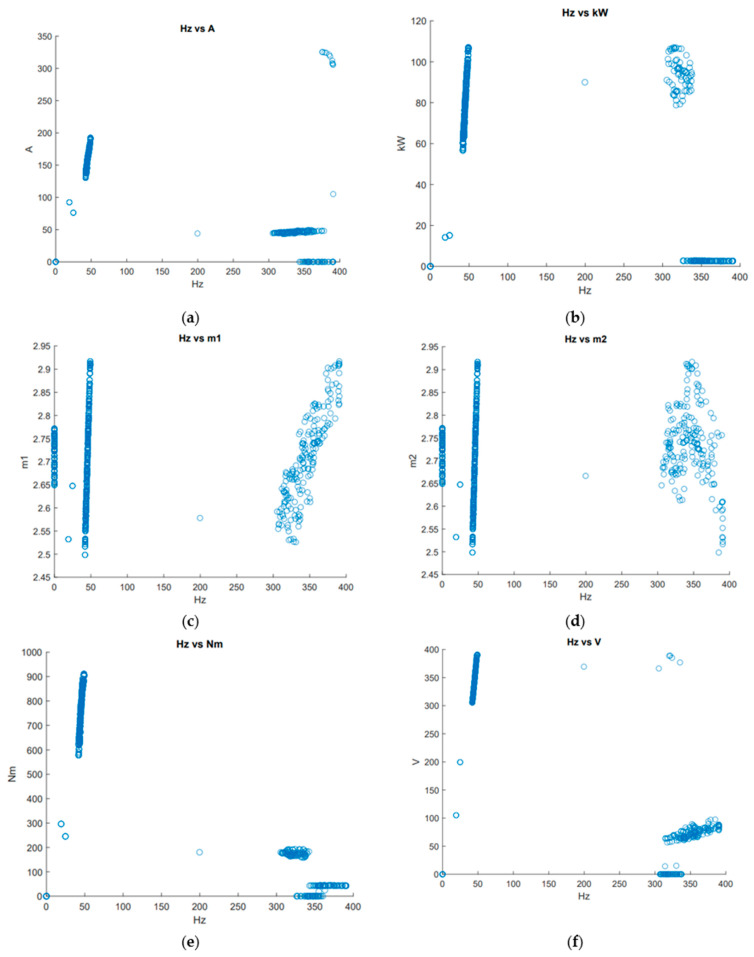
Data on pump operation: (**a**) current (A), (**b**) power (kW), (**c**) sewage level in Chamber A (m1), (**d**) sewage level in Chamber B (m2), (**e**) torque (Nm), and (**f**) voltage (V) depending on frequency (Hz).

The failure-free time estimation problem of the pump is investigated using computer software: MS Excel Solver and GA written in C++ language. The objective is to estimate the failure-free time of the main pump, taking into account the pump condition data. Data on pump operation, including (a) current measured in A, (b) power (kW), (c) sewage level in Chamber A (m1), (d) sewage level in the Chamber B (m2), (e) torque (Nm), and (f) voltage (V) depending on frequency (Hz) were collected for the monthly operation of the main pump (Figure 5). The number of chambers and the equipment used cannot affect the failure-free operation of pumps because the chambers are connected.

### 4.1. Regression Coefficients Estimation Using MS Excel

The presented model is verified with MS Excel. The following hypotheses are tested:

- the failure-free time is proportional to variables Pr_i_ and Ve_i_ taking into account data collected for the main pump and 9 events.
- the failure-free time is proportional to variables m_i_, Pr_i_, Ve_i_, Fy_i_, Ct_i_, Te_i_, and FT_i_ considering data collected for the main pump in 10 days.
- the failure-free time is proportional to variables m_i_, Pr_i_, Ve_i_, Ct_i_, Te_i_, and FT_i_ considering data collected for the main pump in 5 days,
- the failure-free time is proportional to variables m_i_, Pr_i_, Ve_i_, Fy_i_, Ct_i_, Te_i_, and FT_i_ considering data collected for the main pump in 5 days,
- the failure-free time is proportional to variables m_i_, Pr_i_, Ve_i_, Fy_i_, Ct_i_, Te_i_, and FT_i_ considering data collected for the third pump in 5 days,
- the failure-free time is proportional to variables m_i_, Pr_i_, Ve_i_, Ct_i_, Te_i_, and FT_i_ considering data collected for the third pump in 5 days,
- the failure-free time is proportional to variables m_i_, Pr_i_, Ve_i_, Fy_i_, Ct_i_, Te_i_, and FT_i_ considering data collected for two types of pumps (the main and the third) in 5 days.

Taking into account historical data on Pr_i_ and Ve_i_ collected for the main pump for n = 9 failures, the Solver estimates values of unknown regression coefficients b_0_, …, b_6_. The achieved regression formula is (14):

(14) $$9\cdot 0.005588+0.355141\overset{n}{\underset{i=1}{\sum }}{\mathrm{Pr}}_{i}+1.706199\overset{n}{\underset{i=1}{\sum }}{\mathrm{Ve}}_{i}=\overset{n}{\underset{i=1}{\sum }}{\mathrm{FT}}_{i}$$

Under the constraints (15, 16):

(15) $$b_{0}\overset{n}{\underset{i=1}{\sum }}{\mathrm{Pr}}_{i}+b_{1}\overset{n}{\underset{i=1}{\sum }}{\mathrm{Pr}}_{i}^{2}+b_{2}\overset{n}{\underset{i=1}{\sum }}{\mathrm{Pr}}_{i}\cdot {\mathrm{Ve}}_{i}=\overset{n}{\underset{i=1}{\sum }}{\mathrm{Pr}}_{i}\cdot {\mathrm{FT}}_{i}$$

(16) $$b_{0}\overset{n}{\underset{i=1}{\sum }}{\mathrm{Ve}}_{i}+b_{1}\overset{n}{\underset{i=1}{\sum }}{\mathrm{Ve}}_{i}\cdot {\mathrm{Pr}}_{i}+b_{2}\overset{n}{\underset{i=1}{\sum }}{\mathrm{Ve}}_{i}^{2}=\overset{n}{\underset{i=1}{\sum }}{\mathrm{Ve}}_{i}\cdot {\mathrm{FT}}_{i}$$

The estimated values of the parameters of Equation (14) and constraints (15, 16) cannot be accepted since the multiple determination coefficient, R^2^ = 0.000006, and the standard deviation is rather high, s = 371.128 min. The average failure-free time depends to the greatest extent on the voltage variable (Ve_i_) since the value of b_2_ is the highest (1.706199) compared with the value of b_1_.

Taking into account 54 historical observations on m_i_, Pr_i_, Ve_i_, Fy_i_, Ct_i_, Te_i_, and FT_i_ for the main pump collected during 10 days, the Solver estimates values of regression coefficients (17):

(17) $$\begin{array}{c} 54\cdot 0.00128+0.003325\overset{n}{\underset{i=1}{\sum }}m_{i}+0.07901\overset{n}{\underset{i=1}{\sum }}{\mathrm{Pr}}_{i}+0.379\overset{n}{\underset{i=1}{\sum }}{\mathrm{Ve}}_{i}+0.05243\overset{n}{\underset{i=1}{\sum }}{\mathrm{Fy}}_{i}+0.1823\overset{n}{\underset{i=1}{\sum }}{\mathrm{Ct}}_{i}\\ +\;0.81196\overset{n}{\underset{i=1}{\sum }}{\mathrm{Te}}_{i}=\overset{n}{\underset{i=1}{\sum }}{\mathrm{FT}}_{i} \end{array}$$

The estimated values of the parameters of Equation (17) are adequate for the main pump since the multiple determination coefficient R^2^ equals 0.96, and the standard deviation is 29.1058 min. The high value of parameter b_6_ (0.81196) means that the value of failure-free time is high relative to Te_i_ (Nm). The average failure-free time depends to the greatest extent on the torque data. The second most related variable is Ve_i_ since the value of b_3_ is higher (0.379) compared with the values of b_1_, b_2_, b_4_, and b_5_.

Taking into account 30 historical observations on m_i_, Pr_i_, Ve_i_,Ct_i_, Te_i_, and FT_i_ for the main pump collected during 5 days, the Solver Appendix estimates values of regression coefficients (18):

(18) $$\begin{array}{c} 30\cdot 0.001039+0.00271919\overset{n}{\underset{i=1}{\sum }}m_{i}+0.07224642\overset{n}{\underset{i=1}{\sum }}{\mathrm{Pr}}_{i}+0.3239\overset{n}{\underset{i=1}{\sum }}{\mathrm{Ve}}_{i}+0\overset{n}{\underset{i=1}{\sum }}{\mathrm{Fy}}_{i}+0.159429\overset{n}{\underset{i=1}{\sum }}{\mathrm{Ct}}_{i}\\ +\;0.7268277\overset{n}{\underset{i=1}{\sum }}{\mathrm{Te}}_{i}=\overset{n}{\underset{i=1}{\sum }}{\mathrm{FT}}_{i} \end{array}$$

The estimated values of the parameters of Equation (18) are adequate for the main pump since the R^2^ = 0.08 and the standard deviation s = 371.75 min. The high value of parameter b_6_ (0.7268277) means that the value of failure-free time is high relative to Te_i_ (m). The second most related variable is Ve_i_.

Taking into account 45 historical observations on m_i_, Pr_i_, Ve_i_, Fy_i_, Ct_i_, Te_i_, and FT_i_ for the third pump collected during 5 days, the Solver Appendix estimates values of regression coefficients (19):

(19) $$\begin{array}{c} 45\cdot 0.00192+0.005037\overset{n}{\underset{i=1}{\sum }}m_{i}+0.097839\overset{n}{\underset{i=1}{\sum }}{\mathrm{Pr}}_{i}+0.45264\overset{n}{\underset{i=1}{\sum }}{\mathrm{Ve}}_{i}+0.063212\overset{n}{\underset{i=1}{\sum }}{\mathrm{Fy}}_{i}+0.2218254\overset{n}{\underset{i=1}{\sum }}{\mathrm{Ct}}_{i}\\ +\;1.01743\overset{n}{\underset{i=1}{\sum }}{\mathrm{Te}}_{i}=\overset{n}{\underset{i=1}{\sum }}{\mathrm{FT}}_{i} \end{array}$$

The estimated values of the parameters of Equation (19) are not adequate for the third pump since the multiple determination coefficient R2 = 1.28, and the standard deviation s = 547.72 [mins]. The high value of parameter b_6_ (1.01743) means that the value of failure-free time is most related to FT_i_ (Nm). The average failure-free time depends to the greatest extent on the torque data. The second most related variable is Ct_i_ since the value of b_3_ is higher (0.45264) compared with the values of b_1_, b_2_, b_4_, and b_5_.

Let us consider 45 historical observations on M_i_, Pr_i_, Ve_i_, Ct_i_, Te_i_, and FT_i_ for the third pump collected during 5 days, the Solver Appendix estimates values of regression coefficients (20):

(20) $$\begin{array}{c} 45\cdot 0.00192+0.005053\overset{n}{\underset{i=1}{\sum }}m_{i}+0.0981403\overset{n}{\underset{i=1}{\sum }}{\mathrm{Pr}}_{i}+0.45403\overset{n}{\underset{i=1}{\sum }}{\mathrm{Ve}}_{i}+0\overset{n}{\underset{i=1}{\sum }}{\mathrm{Fy}}_{i}+0.2225078\overset{n}{\underset{i=1}{\sum }}{\mathrm{Ct}}_{i}\\ +\;1.0205642\overset{n}{\underset{i=1}{\sum }}{\mathrm{Te}}_{i}=\overset{n}{\underset{i=1}{\sum }}{\mathrm{FT}}_{i} \end{array}$$

In addition, the estimated values of the parameters of Equation (20) are not adequate for the third pump since R^2^ =1.29 and s = 548.022 min. The conclusions are the same as in the case of the third pump’s condition data, including Fy_i_.

Finally, consider 75 historical observations for each data on m_i_, Pr_i_, Ve_i_, Ct_i_, Te_i_, and FT_i_ for the first and third pumps collected during 5 days, the Solver Appendix estimates values of regression coefficients (21):

(21) $$\begin{array}{c} 75\cdot 0.001477+0.003871\overset{n}{\underset{i=1}{\sum }}m_{i}+0.08624454\overset{n}{\underset{i=1}{\sum }}{\mathrm{Pr}}_{i}+0.393149\overset{n}{\underset{i=1}{\sum }}{\mathrm{Ve}}_{i}+0\overset{n}{\underset{i=1}{\sum }}{\mathrm{Fy}}_{i}+0.19305147\overset{n}{\underset{i=1}{\sum }}{\mathrm{Ct}}_{i}\\ +\;0.882946\overset{n}{\underset{i=1}{\sum }}{\mathrm{Te}}_{i}=\overset{n}{\underset{i=1}{\sum }}{\mathrm{FT}}_{i} \end{array}$$

The estimated values of the parameters of Equation (21) are adequate for the main pump since R^2^ = 0.69 and s = 467.9 min. The value of failure-free time is highly related to Te_i_ (Nm). Again, the second most related variable is Ve_i_.

The most adequate multiple linear regression Equation (17) was achieved for the main pump and 10 days of collecting data. The value of failure-free time is highly related to Te_i_ (Nm) for all cases of experiments for estimating the regression coefficients. By substituting the data acquired on the 11th at 522 min—m_i_ = 2.828125, Pr_i_ = 74.87999725, Ve_i_ = 369.2999878, Fy_i_ = 46.90000153, Ct_i_ = 144.6000061, and Te_i_ = 686.4000244—into the Equation (17), the predicted failure-free time ${\hat{x}}_{55}$ equals 732.131644 min. The real failure of the main pump occurred on the 12th at 1263 min.

### 4.2. Regression Coefficients Estimation Using the GA

Historical data on both dependent (FT_i_) and independent variables (m_i_, Pr_i_, Ve_i_, Fy_i_, Ct_i_, and Te_i_) to establish the relationship between modes and failure-free times were used. A relationship between modes and failure-free times (2–8) (independent and dependent variables) was described by a chromosome—a vector of regression coefficients. The affinity function corresponded to the adequacy of the presented model to historical data on m_i_, Pr_i_, Ve_i_, Fy_i_, Ct_i_, Te_i_, and FT_i_.

The GA for estimating pump uptime was coded in Borland C++. Computer simulations were performed for a set of key parameters, namely initial population size (chn = {100, 200}), number of generations (in = {70, 140}), and number of mutation points (mn = {1, 2, 3, 4}). The objective of increasing the coefficient of multiple determination was examined for different values of kay parameters.

Let us analyze the case solution where the Solver appendix achieved the best solution: considering 54 historical observations on M_i_, Pr_i_, Ve_i_, Fy_i_, Ct_i_, Te_i_, and FT_i_ for the main pump collected during 10 days. The Solver Appendix estimates values of regression coefficients (17) with the multiple determination coefficient R2 equals 0.96, and the standard deviation is 29.1058 min.

**Figure 6:**
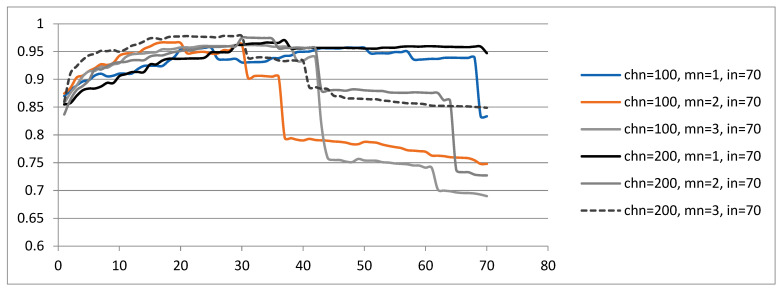
The mean scalar function obtained in each iteration for the simulation described by {chn, mn, in = 70}.

First, the average quality of the population for computer simulations described by {chn, mn, in = 70} is examined (Figure 6). The mean scalar function increases as soon as computer simulations reach approximately 20 iterations. After approximately 30 iterations, the mean scalar function decreases, except for the experiment described by {chn = 200, mn = 1, in = 70}. The mean scalar function improves as the number of mutation points increases. As can be seen, better results are not so much related to the size of chn as to mn.

Table 3 presents the best multiple determination coefficients obtained for the regression coefficients (b_0_, b_1_, …, b_6_) in the simulation described by {chn, mn and in = 70}. The values of R^2^ should be in the range [0, 1]. Therefore, directed selection pressure with four conditions was developed (Figure 4). For example, a solution survives when R^2^ approaches 1 from a pair (parent and offspring) with coefficients greater than or equal to 1 in the first condition. In other cases, a solution survives when R^2^ approaches 1 from a pair (parent and offspring) with coefficients less than 1.

**Figure 7:**
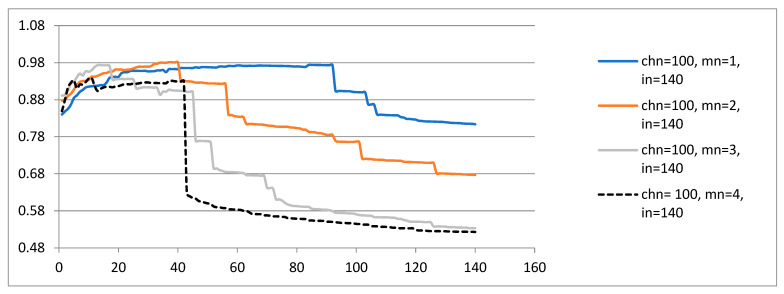
The mean scalar function obtained in each iteration for the simulation described by {chn, mn, in = 140}.

Next, the average quality of the population is examined for computer simulations described by {chn, mn, in = 140} (Figure 7). The same phenomenon is noticed: the mean scalar function increases as soon as computer simulations reach approximately 20 generations. After approximately 40 generations, the mean scalar function decreases. For the experiment described by {chn = 100, mn = 1, in = 140}, the GA works according to its idea of improving the genetic material from generation to generation only after 100 iterations. The phenomenon of improving the mean scalar function with the increase in mn was clearly visible for the computer simulations described by {chn, mn, in = 140}. The conclusion was proved that better results are not so much related to chn as to mn.

A comparison of the average quality of the population for computer simulations described by both {chn, mn, in = 70} (Figure 6) and {chn, mn, in = 140} (Figure 7) allows us to draw the following conclusion: a greater number of mutation points {mn = 3.4} and iterations {in = 140} produce better results.

Table 4 presents the best R^2^ obtained for the regression coefficients (b_0_, b_1_, …, b_6_) in the simulation described by {chn, mn and in = 140}. R^2^ approaches 1 with more mutation points. More iterations are required for GA to find the right solution, even using the directed selection pressure.

Although the best coefficient of multiple determination was very good, R(x_4_)^2^ = 0.99 (Table 4), the standard deviation was very high (614); hence, the achieved equation does not fit well with the historical data. The average failure time of the main pump depends to the greatest extent on Pr_i_, Fy_i_, and Ct_i_:

(22) $$\begin{array}{c} 54\cdot \left(-5.99356\right)+0.01273\overset{n}{\underset{i=1}{\sum }}M_{i}+5.01244\overset{n}{\underset{i=1}{\sum }}{\mathrm{Pr}}_{i}+0.00488\overset{n}{\underset{i=1}{\sum }}{\mathrm{Ve}}_{i}+5.00025\overset{n}{\underset{i=1}{\sum }}{\mathrm{Fy}}_{i}\\ +\;5.00061\overset{n}{\underset{i=1}{\sum }}{\mathrm{Ct}}_{i}+0.0195\overset{n}{\underset{i=1}{\sum }}{\mathrm{Te}}_{i}=\overset{n}{\underset{i=1}{\sum }}{\mathrm{FT}}_{i} \end{array}$$

The next section contains further analyses and discussion related to the research on the application of the GA.

## 5. Discussion of the Results of the GA Experiments

The presented method is useful for managers to obtain an accurate forecast. A high selection procedure is given for the smallest s of the obtained forecasts (9). On the contrary, in Section 4, the results were obtained with R^2^ (10) for selection pressure.

**Figure 8:**
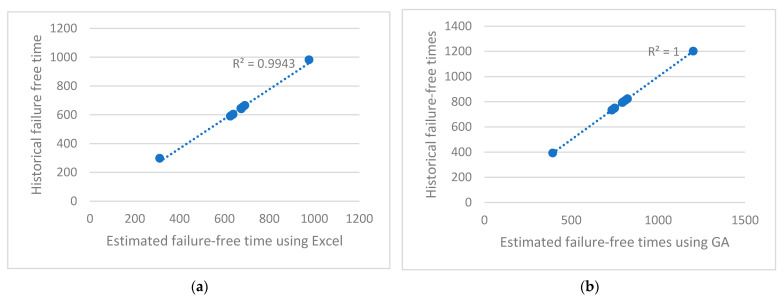
Variation of estimated failure-free time (minutes) depending on historical failure-free times achieved using (**a**) Excel (17) and (**b**) GA (23).

Six computer simulations were carried out for the values of key parameters: chn = 100, in = 100, and mn = {3, 4, 5} (Table 5). Coefficient R^2^ is at a maximum for (23) obtained using the GA (Figure 8). Although the best R(x_6_)^2^ = 1, s is still very high (571). The average failure time of a main pump depends to the greatest extent on Ve_i_, Ct_i_, and Te_i_, according to (23):

(23) $$\begin{array}{c} 54\cdot 8+0.00345\overset{n}{\underset{i=1}{\sum }}M_{i}+0.000632\overset{n}{\underset{i=1}{\sum }}{\mathrm{Pr}}_{i}+0.0435\overset{n}{\underset{i=1}{\sum }}{\mathrm{Ve}}_{i}+8.1e-05\overset{n}{\underset{i=1}{\sum }}{\mathrm{Fy}}_{i}+0.0389\overset{n}{\underset{i=1}{\sum }}{\mathrm{Ct}}_{i}\\ +\;0.0923\overset{n}{\underset{i=1}{\sum }}{\mathrm{Te}}_{i}=\overset{n}{\underset{i=1}{\sum }}{\mathrm{FT}}_{i} \end{array}$$

Finally, we performed simulations for a limited range of the J gene from [0, 2] to reduce the solution space and, thus, increase the chances of achieving a better solution. Two experiments were run for {chn = 100, mn = 3, in = 100}, and the GA achieved linear regression coefficients (24, 25):

(24) $$\begin{array}{c} 54\cdot 2+0.000148\overset{n}{\underset{i=1}{\sum }}M_{i}+6.00005\overset{n}{\underset{i=1}{\sum }}{\mathrm{Pr}}_{i}+0.00345\overset{n}{\underset{i=1}{\sum }}{\mathrm{Ve}}_{i}+0.00229\overset{n}{\underset{i=1}{\sum }}{\mathrm{Fy}}_{i}+0.0924\overset{n}{\underset{i=1}{\sum }}{\mathrm{Ct}}_{i}\\ +\;0.0449\overset{n}{\underset{i=1}{\sum }}{\mathrm{Te}}_{i}=\overset{n}{\underset{i=1}{\sum }}{\mathrm{FT}}_{i} \end{array}$$

(25) $$\begin{array}{c} 54\cdot 1.00056+0.00338\overset{n}{\underset{i=1}{\sum }}M_{i}+4.64E-05\overset{n}{\underset{i=1}{\sum }}{\mathrm{Pr}}_{i}+0.00833\overset{n}{\underset{i=1}{\sum }}{\mathrm{Ve}}_{i}+8.63e-05\overset{n}{\underset{i=1}{\sum }}{\mathrm{Fy}}_{i}+1.00122\overset{n}{\underset{i=1}{\sum }}{\mathrm{Ct}}_{i}\\ +\;1.00795\overset{n}{\underset{i=1}{\sum }}{\mathrm{Te}}_{i}=\overset{n}{\underset{i=1}{\sum }}{\mathrm{FT}}_{i} \end{array}$$

**Figure 9:**
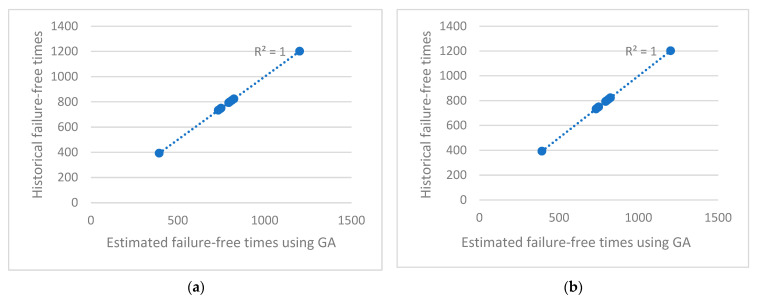
Variation of estimated failure-free time (minutes) depending on historical failure-free times achieved using (**a**) GA (24) and (**b**) GA (25).

Coefficient R^2^ achieved the maximum value in both experiments (Figure 9). The value s for the linear regression (24) is 223 min. The value s for the linear regression (25) is 157 min. The average failure time of the main pump depends to the greatest extent on Pr_i_ in the case of (24). The average failure time of the main pump depends to the greatest extent on Ct_i_ and Te_i_, according to (25).

Managers of the sewage treatment plant may substitute new data for sewage level (m), power (kW), voltage (V), frequency (Hz), current (A), and torque (Nm) into Equation (25) in order to estimate the future failure-free time. The predicted failure-free time of the main pump is 626 min.

As a result of the analysis of the obtained results (Figure 8 and Figure 9), the following conclusions can be drawn:

- The agreement of the regression line with the empirical data is very good (even 99–100%), while the GA obtained a slightly better fit (R^2^ = 1) compared with Excel (R^2^ = 0.9943).
- In both graphs, the estimated repair time is very sensitive to an increase in historical time: a relatively small increase in historical time results in a stronger increase in estimated repair time.
- Further research is needed taking into account more days of data collection to support the conclusion that the time between failures of the main pump depends on current and torque.

## 6. Conclusions

Planning the operation and repair of pumps in a wastewater treatment plant is a very important activity due to the serious consequences of potential failures, including environmental contamination and high costs. It was assumed that the tool for forecasting the time of failure-free operation can be based only on historical data collected from the SCADA system, without installing additional sensors and measuring instruments or interfering with SCADA and the current operation of the sewage treatment plant. Integration of the developed method with online data acquisition using SCADA consists of continuous substitution of the collected data into the resulting equation and calculation of the pump’s failure-free operation time of the sewage treatment plant.

As a result of the analysis of related topics, it was decided to use multiple linear regression in MS Excel and a specially developed GA. It was found that the GA simulation results were slightly better than those using MS Excel. The parameters obtained with the help of GA better described the relationship of the data: dependent historical (failure-free time) and independent (the main pump condition). The proposed GA modifications helped to obtain the optimal solution, taking into account the coefficient of multiple determination.

Wastewater treatment plant managers obtained the predictive equation to predict the failure-free operation of the pumps. The conducted analysis allowed for drawing the conclusion that the time between failures of the main pump depends to the greatest extent on current and torque. The presented method of condition-based maintenance can be easily applied to a variety of equipment.

In the future, various artificial intelligence methods will be explored to compare with GA to produce a linear regression with a lower standard deviation between empirical and historical data. It is also planned to establish standards for variables describing the operating conditions of pumps using the six-sigma method.

## Figures and Tables

**Table 1:** DNA library of genes for each sub-chromosome generation.

$V=\left[-1,1\right]$	← a gene for a sign is generated at random
$D=\left[0,1\right]$	← a gene for yes or no decisions for integer values is generated at random
$J=\left[1,\ldots ,20\right]$	← a gene for a coefficient value before the decimal point is generated at random
$C=\left\{\begin{array}{c} 0\;if\;D=1\\ 1\;if\;D=0\;or\;1 \end{array}\right.$	← a gene for a decision yes or no for fractional values is generated
$E=\left[0.1,0.01,0.001,0.0001,0.00001\right]$	← a gene for a number of zeros after the decimal point is generated
$F=\left[1,\ldots ,999\right]$	← a gene for a coefficient value after zeros is generated

**Table 2:** The example of a chromosome generated from the DNA library.

$x_{j,n}$	V	D	J	C	E	F
***b*** **0**	−1	1	12	1	0.0001	161
***b*** **1**	−1	1	10	0	0.0001	260
***b*** **2**	+1	0	3	1	0.001	35
***b*** **3**	+1	0	9	1	0.0001	189
***b*** **4**	−1	1	3	1	0.0001	33
***b*** **5**	+1	0	2	1	0.001	300
***b*** **6**	+1	0	1	1	0.01	271

**Table 3:** Multiple determination coefficient obtained for the best regression coefficients (b_0_, b_1_, …, b_6_) in the simulation described by {chn, mn, in = 70}.

chn	mn	in	$R{\left(x_{\eta }\right)}^{2}$	b_0_	b_1_	b_2_	b_3_	b_4_	b_5_	b_6_
100	1	70	2.24673	−3.917	11.0094	0.0521	0.01009	7.115	0.0799	0.0102
100	2	70	2.24673	13.0921	13.0753	0.001266	0.0966	16.0001	−8.99182	0.1275
100	3	70	1.39895	−16.9932	−17.9816	20.0783	0.0104	8.00675	0.000423	0.0359
200	1	70	9.89124	−9.98925	0.01075	0.094	0.0112	0.1179	0.1015	0.0959
200	2	70	1.36501	−13.9895	16.0001	18.0098	0.0611	9.83 × 10^−5^	0.1014	0.01156
200	3	70	2.71901	−18.9997	0.0797	1.83 × 10^−5^	0.000544	−14.9996	2.08 × 10^−5^	4.00326

**Table 4:** Multiple determination coefficients obtained for the best regression coefficients (b_0_, b_1_, …, b_6_) in the computer simulation described by {chn, mn, in = 140}.

chn	mn	in	$R{\left(x_{\eta }\right)}^{2}$	b_0_	b_1_	b_2_	b_3_	b_4_	b_5_	b_6_
100	1	140	2.23819	−14.9844	14.0132	5.01014	0.1517	0.01482	4.1295	0.01437
100	2	140	1.13929	−11.9967	12.0448	20.0001	0.00669	0.000757	0.0286	0.00051
100	3	140	1.00739	−7.9868	0.0068	20	0.00341	−4.8944	7.67 × 10^−5^	3.88 × 10^−5^
100	4	140	0.999998	−5.99356	0.01273	5.01244	0.00488	5.00025	5.00061	0.019

**Table 5:** Multiple determination coefficients obtained for regression coefficients (b_0_, b_1_, …, b_6_) in the computer simulation described by {chn = 100, mn, in = 100}.

chn	mn	in	$R{\left(x_{\eta }\right)}^{2}$	b_0_	b_1_	b_2_	b_3_	b_4_	b_5_	b_6_
100	3	100	0.996116	−5.9056	−12.9963	0.00081	8.18 × 10^−5^	0.0037	9.00895	0.000855
100	4	100	0.999721	−2	0.00743	0.00889	0.0264	10.0047	0.00518	0.000963
100	5	100	0.970872	18	0.00662	1.17 × 10^−5^	3.01 × 10^−5^	9.85 × 10^−5^	0.000171	−0.999414
100	3	100	1	−6.9345	0.000695	0.00182	0.00626	0.0059	5.00087	0.0825
100	4	100	1	15	0.00606	0.096	2.79 × 10^−5^	0.000912	0.000926	0.00341
100	5	100	1	8	0.00345	0.000632	0.0435	8.1 × 10^−5^	0.0389	0.0923

## Data Availability

Not applicable.
